# The protective effects of plasma gelsolin on stroke outcome in rats

**DOI:** 10.1186/2040-7378-3-13

**Published:** 2011-11-02

**Authors:** Huong T Le, Aaron C Hirko, Jeffrey S Thinschmidt, Maria Grant, Zhimin Li, Joanna Peris, Michael A King, Jeffrey A Hughes, Sihong Song

**Affiliations:** 1 Department of Pharmaceutics, University of Florida College of Pharmacy, Gainesville, FL 32610, USA; 2 Department of Pharmacology & Therapeutics, University of Florida College of Medicine, Gainesville, FL 32610, USA; 3 Department of Pharmacodynamics, University of Florida College of Pharmacy, Gainesville, FL 32610, USA; 4 Department of Veterans Affairs Medical Center, Gainesville, FL 32602, USA; 5 Medco School of Pharmacy, Fairleigh Dickinson University, Madison, NJ 07940, USA

**Keywords:** ischemic stroke, plasma gelsolin, protective effect, endothelin-1 induced MCAO

## Abstract

**Background:**

To date, recombinant tissue plasminogen activator (rtPA) is the only approved drug for ischemic stroke. It is intravenously administered functioning as a thrombolytic agent and is used to obtain reperfusion of the affected area of the brain. Excitotoxicity, inflammation and apoptosis are all involved in delayed neuronal death following stroke and offer multiple opportunities to intervene with neuroprotective agents. Gelsolin (GSN) is an actin- and calcium-binding protein mediating the disassembly of actin filaments and activity of calcium channels. It also functions as a regulator of apoptosis and inflammatory responses. This study tests the hypothesis that increasing the concentration of the form of GSN known as plasma GSN (pGSN) near an infarct will provide neuroprotection following ischemic stroke.

**Methods:**

We induced middle cerebral artery occlusion (MCAO) in male rats via intracranial injection of endothelin-1 (ET-1), a potent vasoconstrictor, and then treated with local delivery of pGSN. Whole brain laser Doppler perfusion imaging was performed through the skull to assess MCAO effectiveness. Cylinder and vibrissae tests evaluated sensorimotor function before and 72 h after MCAO. Infarct volumes were examined 72 h after MCAO via 2, 3, 5-triphenyltetrazolium chloride (TTC) assay.

**Results:**

Estimates of relative cerebral perfusion were significantly decreased in all groups receiving MCAO with no differences detected between treatments. Despite equivalent initial strokes, the infarct volume of the pGSN treatment group was significantly reduced compared with the untreated MCAO rats at 72 h. ET-1 induced significant deficits in both cylinder and vibrissae tests while pGSN significantly limited these deficits.

**Conclusion:**

Gelsolin could be a promising drug for protection against neurodegeneration following ischemic stroke.

## Introduction

Stroke or brain attack occurs when the blood supply to the brain is interrupted, usually because a blood vessel is blocked by a clot or loses structural integrity permitting hemorrhage. The disease is not subject to a particular race or ethnic group [1]. In 2009, 795, 000 strokes occurred in the United States, i.e. a stroke occurs once every 40 seconds and a death occurs every 4 minutes [2]. According to the Centers for Disease Control and Prevention (CDC), the total cost of stroke was $68.9 billion and the number is expected to rise. Of all strokes, 87% are ischemic [2]. Currently, recombinant tissue plasminogen activator (rtPA) is the only FDA-approved therapeutic agent for ischemic stroke. rtPA is effective only if intravenously administered within 3 to 4.5 h of stroke onset, and can have adverse neurotoxic effects even with proper use [3]. The drug can only be used within a narrow time window after a stroke begins and only about two percent of stroke patients are able to access rtPA therapy. Therefore, development of new agents for stroke is essential.

The mechanisms involved in stroke injury and repair are extremely complex, involving excitotoxicity and necrotic cell death occurring within minutes of stroke onset [4]. As well, there is increasing evidence showing that genetically programmed cell death during post-ischemic tissue inflammation (that can last days to weeks) has a detrimental effect [5,6]. Therefore, therapeutic strategies targeting that delay or dampening inflammatory responses could inhibit the progression of the tissue damage and improve the overall outcome of stroke.

Gelsolin (GSN) is a ubiquitous [7,8] actin filament-severing, capping and actin nucleation protein of eukaryotes. Originally described as an actin-binding protein, GSN exists in both intracellular (cytoplasmic protein, cGSN) and extracellular (a secreted protein or plasma gelsolin, pGSN) forms. pGSN, also known as brevin and actin-depolymerizing factor, consists of a single 755-amino acid polypeptide chain (84 kDa) including a 25- amino acid N-terminal extension [9] that distinguishes it from cGSN (82 kDa). Most cells secrete pGSN, however smooth, skeletal and cardiac muscle cells produce larger amounts of pGSN [7]. The plasma concentration of pGSN is 200-300 mg/L [10-12] and isolated human and rabbit pGSN have a plasma half-life of 2.3 days [13]. Because pGSN derives from muscle tissue, it must pass through interstitial fluid of the extracellular matrix to localize in the blood. pGSN also exists in human cerebrospinal fluid (CSF) [14]. Although certain functions for the intracellular isoforms have been described, the function(s) of the plasma isoforms remain unclear. The high affinity of pGSN for filamentous actin (F-actin) (K_a _> 10^9^/mol/L) [15] suggests that its physiological function is likely related to its actin-binding properties. pGSN may scavenge actin leaked from injured tissue and limit subsequent damage instigated by extracellular filamentous actin [16]. Studies have shown that large amounts of F-actin could potentially increase the blood viscosity and perturb blood flow through the microvasculature [17].

It is also well established that pGSN levels decrease in blood in acute inflammation conditions that involve tissue damage [18-21]. Consistent with the idea that pGSN is not only a biomarker for inflammation but also an important protective factor, repletion of pGSN in a mouse model of endotoxemic sepsis led to solubilization of circulating actin aggregates and significantly reduced mortality in mice [22]. GSN knockout mice neurons are vulnerable to glucose/oxygen deprivation, and pharmacological brain actin depolymerization restored resistance to ischemic stroke in knockout mice [23]. The knockout mice results could not determine which endogenous form of gelsolin is responsible, or whether gelsolin in or near the infarct mediates neuroprotective effects. Gelsolin-overexpressing transgenic mice demonstrate neuroprotection against experimental stroke [23], but it is not known whether these effects are mediated by pGSN or cGSN, or whether it is GSN near the infarct that mediates the protection.

To test the hypothesis that proximal administration of pGSN can antagonize stroke pathology, we induced transient middle cerebral artery occlusion (tMCAO) in male rats via intracranial injection of ET-1, a potent vasoconstrictor, and post-treated with discrete brain injection of pGSN. Cylinder and vibrissae tests were used to examine sensorimotor function before and 72 h after MCAO to assess functional deficits. Whole brain laser Doppler perfusion imaging was performed through the skull to verify MCAO effectiveness. Infarct volumes were examined 72 h after MCAO using 2, 3, 5-triphenyltetrazolium chloride (TTC) assay.

## Materials and methods

### Materials

Endothelin-1 (1-39) rat (ET-1), purchased from American Peptide Company, Inc. (CA, U.S.A.), was dissolved in sterile phosphate buffered saline (PBS) to make a stock concentration of 80 μM. The stock solution was stored at -20°C. ET-1 was thawed, centrifuged and placed on ice until ready to inject. Human plasma gelsolin (pGSN) was a generous gift from Critical Biologics Corporation (MA, U.S.A.) Artificial cerebrospinal fluid (aCSF) was obtained from Fisher Scientific, Inc. (PA, U.S.A.)

### Animal model

Experiments were conducted on 19 male Sprague-Dawley rats weighing from 210-270 g. Animals were randomly divided into five groups: two groups of three for laser Doppler measurement, two groups of five for experimental ischemic stroke by ET-1 plus pGSN or control injections, with evaluation of behavior and brain damage, a group of three for artificial cerebrospinal fluid (aCSF) injection. Two animals in the group of five injected with ET-1 alone were eliminated from the study: one died on day 2 and one did not show any movement in behavioral studies. One animal was eliminated from the ET-1 plus pGSN group since we could not define the infarction area. The exclusion rate was 3/13 for the treatment study and 0/6 for the laser Doppler study. All procedures were implemented according to a protocol approved by the University of Florida *Institutional Animal Care and Use Committee *(*IACUC*).

A model of reversible focal ischemia using ET-1 was used as previously described [24,25]. When ET-1 is injected proximal to the middle cerebral artery (MCA), it acts on vascular smooth muscle cell receptors ET_A _and ET_B _[26], causing arterial constriction and reduced blood flow to areas in the brain supplied by the ipsilateral MCA (anterolateral neocortex, cortical areas, and caudate nucleus). Anesthesia was initiated with isoflurane (5% in O_2 _and maintained with 1.5-2.5% in O_2_). Body temperature was maintained between 36.5-37.5°C throughout surgery using a water-jacketed heating blanket. The animals were placed in a stereotaxic frame (Braintree Scientific, Inc., MA, U.S.A.) and secured in the flat skull position. A midline incision was made spanning the bregma and lambda landmarks, and a small hole (3 mm i.d) was drilled in the cranium adjacent to the left MCA (coordinates: 0.2 mm anterior, 5.2 mm lateral, and 1 mm dorsal to the bottom of the skull). A 27-gauge needle was used to inject 3 μL of ET-1 (240 pmol) at 1 μL/min. After ET-1 injection, the needle was left in place for an additional 3 min before being slowly withdrawn. The system was flushed by DI water and pGSN was loaded in the needle. Approximately, 5-10 min after ET-1 injection, 3 μL of pGSN (3 μg or 35.71 pmol) was injected, after which the needle was again left in place for 3 min, then slowly removed. The incision was closed and animals were kept warm at 37°C until totally recovered from anesthesia.

### Cerebral Perfusion Measurement

In each animal, skin on the scalp was removed and two holes (3 mm i.d) were made above in each hemisphere. ET-1 or ET-1 plus pGSN were injected in the left side of the brain and the other side was used as an internal control. Relative blood flux/perfusion measurements were made using Laser Doppler (MOORLDI, Moor Instrument Ltd, UK) at four different time points: pre-injection, 10-20 min, 30-35 min, and 55-65 min from the ET-1 injection time. The measurements were based on the moving blood in the microvasculature that causes a Doppler frequency shift of the scattered laser light, which is photodetected and then processed to build a color coded map of relative blood flow. A digital camera records a color clinical photograph at the same time, corresponding closely with the blood flow image in size and aspect. The pseudocoloring is relative flux (number of red blood cells multiplied by speed) where "warm" colors indicate relative high flux and "cool" colors represent relative low flux. The 16- bit color scans were made with arbitrarily assigned unit from 0 (lower limit) to 1, 000 or more (upper limit). The scan speed was 10 ms/pixel and the total scan (scan area was about 1.8 cm × 2.3 cm) duration per animal was approximately 10 min. Actual blood flow is impossible to measure with Laser-Doppler flowmetry (LDF), but is highly correlated to flux except at supranormal pressures [27]. Indeed, percentage reductions of cerebral perfusion are actually relative percentage reductions of flow and were calculated using the following formula:

(1) $$\left(\text{Pi}∕\text{Pc}\right)\times 100\%$$

where Pi is flow values at ipsilateral side (injected side) and Pc is flow values at contralateral side (unaffected side); both are measured in perfusion unit (PU)

### Behavioral tests

Behavioral tests used include the limb-use asymmetry (cylinder) and vibrissae-stimulated forelimb placing (vibrissae) for somatosensory asymmetry. Animals were tested before and 72 h post-surgery. The cylinder test examines the level of preference for using the non-impaired forelimb for weight shifting movements during spontaneous vertical exploration. Non-infarcted animals typically use both limbs equally for upright support, but after damage to the motor system, animals show an asymmetric reliance on the less-affected (ipsilateral) limb [28]. Rats were placed in a specially designed transparent cylinder (20 cm i.d, 40 cm height) for 3 min. The cylinder was high enough that the animal could not reach the top edge or escape, but encourages vertical exploration of the glass walls with the forepaws as well as landing (return to floor) activity. Forepaw use during exploration of the rats was scored by analyzing slow-motion playback of digital video recorded from a camera installed over the cylinder, by an experimenter blind to the treatment condition. The fraction of events in which the animal used the ipsilateral (affected), contralateral (un/less affected), or both forelimbs was calculated as a dependent measure of asymmetry.

Vibrissae-stimulated forepaw and extinction placing tests evaluate sensorimotor/proprioceptive deficits. To determine whether an animal has asymmetrical sensorimotor perception, the animal is held by the torso with its forelimbs hanging freely, and then slowly moved laterally toward the edge of the table or countertop until the vibrissae of one side makes contact with the edge. Intact rats quickly place the ipsilateral forepaw on the edge or the surface of the table when the ipsilateral vibrissae brush the table edge. In contrast, rats with damage to the motor system often do not respond to vibrissae stimulation on the affected side (contralateral side). Using Windows Movie Maker 2007 (Microsoft), a treatment-blinded viewer scored digital video records for each side, using the frame rate and the number of frames between vibrissae stimulation and forepaw contact to determine placing response delay intervals.

### Histology

Animals were sacrificed at 72 h for TTC staining. The brains were quickly isolated, placed in cold PBS (0-4°C) for 30 min and then sectioned into 2-mm thick coronal slices using a brain slice matrix (Leica Microsystems, IL, U.S.A.) The tissue sections were held in cold PBS for 3 min before they were incubated in TTC solution (0.05% TTC in PBS) for 30 min at 37°C. The sections were washed three times with PBS (one minute each) and fixed in 0.1 M phosphate buffered formaldehyde (PBF). The sections then were placed on a flatbed scanner (EPSON 1680) to obtain images.

### Infarction Area Analysis

Calibrated digital images of tissue sections were made at 600-dpi scanner resolution with 48-bit color and saved as TIFF files. The infarction areas were quantified by visual thresholding of TTC-labeled (normal) and unlabeled (infarct) tissue, and measurement of each area, using Image J version 5.0 (NIH). Infarct volume was estimated as the product of area on each section and the number of 2 mm sections exhibiting infarct. The ratio of infarct to normal volume was used as dependent measures for evaluating pGSN effects.

### Statistical analysis

All data are expressed as arithmetic means ± s.e.m. Two-way ANOVA (time and treatment) was followed by Bonferroni *post hoc *test to compare behavioral dependent variables between groups. Comparisons of infarct area were made by two-tailed Student's t-test; and p-values less than 0.05 were considered to be significant.

## Results

### Treatment of pGSN did not interrupt ET-1 induced artery contraction

**Figure 1:**
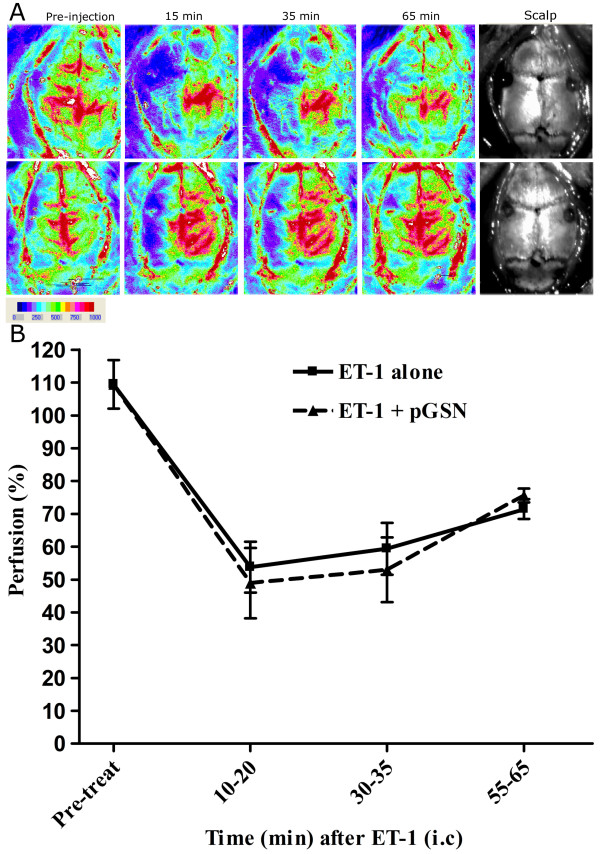
**Perfusion measurements**. **(A) **Color photographs of an animal brain recorded at different time points (pre-injection, 10-20 min, 30-35 min, and 60-65 min from ET-1 injection time) by a digital camera of laser Doppler system, corresponding closely with the blood flow image. Look-up table shows arbitrarily assigned perfusion unit (PU) from the lower limit 0 to upper limit 1, 000 and above. The scalps demonstrate the holes and injection sites. **(B) **Percentage perfusion reduction as a function of time calculated using formula 1. All rats were injected with ET-1 (240 pmol in 3 μL PBS) proximal to the left middle cerebral artery. About 5-10 min after ET-1 injection, pGSN (35.71 pmol in 3 μL saline) was intracranially injected (i.c) at the same site in a group and no injection in the other group. The dotted line represents the average levels in pGSN treated group (N = 3). The solid line represent levels in control group (N = 3). The differences at all-time points were not statically significant.

In order to test the effect of pGSN and ET-1 on MCAO, cohorts (n = 3) of rats were injected with ET-1 to induce transient middle cerebral artery occlusion (MCAO). Approximately 5-10 min after ET-1 injection, pGSN or saline was injected at the same location. The time points of scanning were based on ET-1 injection time. The relative perfusion unit (PU) or blood flow values of animals before injection were in the range of 800-1, 600 PU and the difference between two hemispheres of the brain was not statistically significant. After 20 min following ET-1 injection, the flux values on the injection side dropped to the range of 300-600 PU. The calculations were made using formula 1 described in the methods. Relative flow values showed a rapid decrease to ~ 50% of baseline in all animals and flow remained maximally decreased for 20-30 min after ET-1 injection (Figure 1). ET-1 injection resulted in a drop in perfusion immediately after injection regardless of pGSN injection at the same location. Reperfusion was observed in both groups after ~ 60 min. These results indicate that pGSN injection did not interrupt the induction of artery contraction by ET-1.

### Treatment of pGSN significantly reduced ET-1 induced behavioral deficits

To test the protective effect of pGSN, behavioral studies were conducted. Initially, five rats were randomly assigned into ET-1 injection alone or ET-1 plus pGSN group. In the ET-1 only group, one rat died and one rat was paralyzed after ET-1 injection and was euthanized immediately. In ET-1 plus pGSN treated group, one rat did not show any infarction by TTC staining and was excluded. Therefore, three animals in ET-1 group and four animals in ET-1 plus pGSN group were examined for behavioral deficits and brain damage.

#### Cylinder test

**Figure 2:**
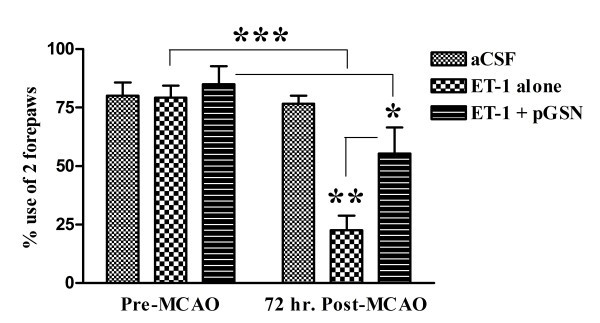
**Cylinder test**. Rats were placed in a transparent cylinder for 3 min. Animal forepaws used during exploration were scored. Each bar represents the average fraction of using both forepaws in the indicated group. ET-1 group, N = 3, ET-1 + pGSN, N = 4, *: p-value < 0.05; **, p-value < 0.01, ***, p-value < 0.001.

As shown in Figure 2, ET-1 induced MCAO resulted in profound impairment of contralateral forepaw function three days following injection. ET-1-treated animals showed significant reduction of symmetrical forepaw use during wall exploration (from approximately 80% to 20%), while artificial cerebrospinal fluid (aCSF) injection had no effect. In the pGSN treatment group, the percentage of symmetrical forepaw use was significantly higher than the control group (20% vs. 70%, p-value < 0.01). The number of attempts to explore the wall was also lower than before surgery (data is not shown). These results indicate that pGSN treatment significantly prevented MCAO-induced damage to the motor system.

#### Vibrissae test

**Figure 3:**
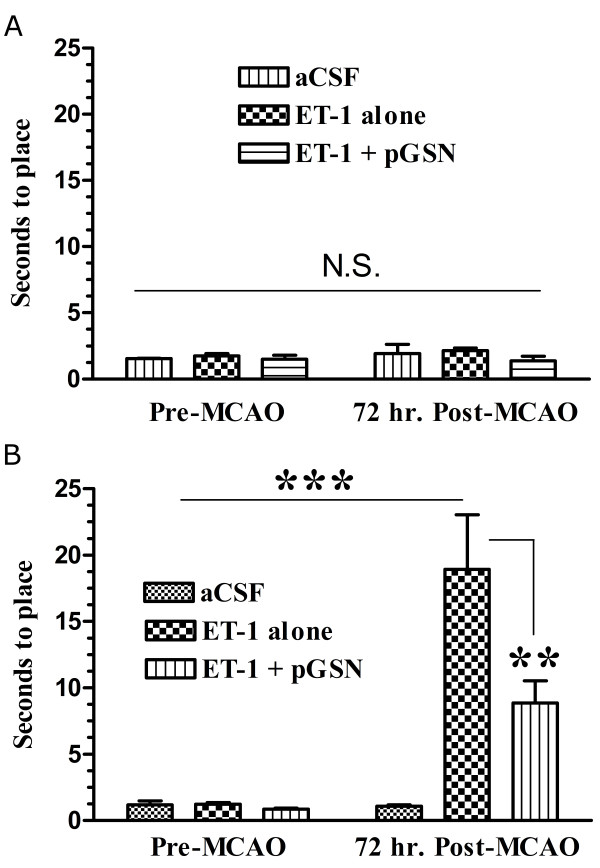
**Vibrissae test**. **(A) **Time in seconds to ipsilateral forepaw placement on the countertop. **(B) **Time in seconds to contralateral forepaw placement on the countertop, ET-1 group, N = 3, ET-1 + pGSN, N = 4, **: p-value < 0.01.

To test the effect of pGSN on sensorimotor system, we also performed vibrissae testing. As expected, vibrissae-stimulated placing of the ipsilateral forepaw was not affected 3 days after experimental ischemic stroke (Figure 3A). Contralateral forepaw placing was significantly slowed (from 1 s to 18 s) in the control (ET-1 alone) group (Figure 3B). Intriguingly, pGSN treatment significantly reduced the time of contralateral forepaw placing compared to control group (18 s vs. 9 s, p-value < 0.01). These results indicated that pGSN treatment significantly prevented the loss of sensorimotor function induced by MCAO.

### Treatment of pGSN reduced MCAO induced brain damage

**Figure 4:**
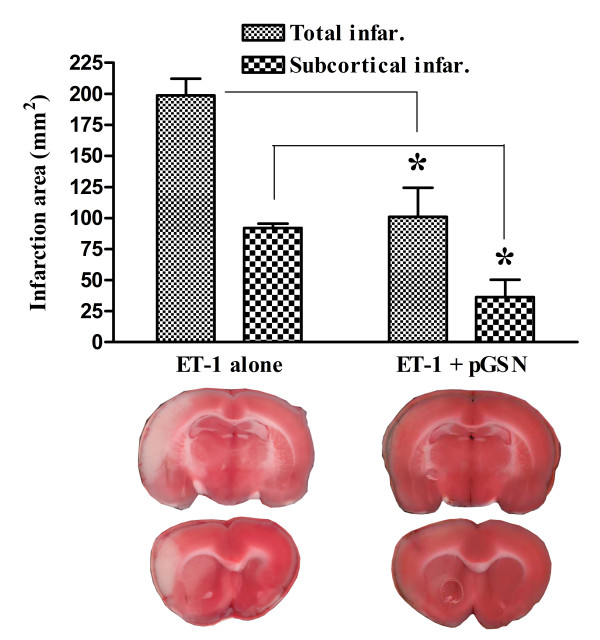
**Infarction area labeled for mitochondrial activity (triphenyltetrazolium chloride, TTC)**. ET-1-alone group N = 3, pGSN treatment group, N = 4. *, p-value < 0.05. Four TTC-staining slices from two animals, one for each group, at the area received most of blood from MCA. Off-white color areas show death tissues while red areas indicate vital tissues.

In the control rats, ET-1 produced large and reproducible unilateral infarcts that involved the rostro-central dorsolateral cortex and basal ganglia, corresponding to the full extent of the MCA territory. The infarction volume in the pGSN-treatment group was reduced by 49% compared to the control group (Figure 4). Sparing was observed in both cortical and subcortical structures.

## Discussion

The current study reports the first time use of pGSN as a protein drug to reduce injury after transient local ischemic stroke. We demonstrate that pGSN can improve sensorimotor recovery in conjunction with substantial reduction in infarct volume present three days after stroke.

Transient middle cerebral artery occlusion (tMCAO) induced by ET-1, a potent vasoconstriction peptide, decreased relative cerebral blood flow in brain tissue served by the MCA by 50% in all groups. In previous studies, injection of ET-1 adjacent to MCA has been shown to reduce blood flow 30-75% in the region supplied by the artery including cortex and basal ganglia, and to produce subsequent ischemic neuropathology in these regions of the brain [24,25,29].

Our results demonstrate that after 10-20 min following ET-1 intracranial injection, the ipsilateral cerebral relative blood flow decreased approximately 50% in all groups of animals (Figure 1), indicating that all animals initially have comparable ischemic strokes and that pGSN did not reduce the magnitude of the original ischemic event, but limited the subsequent degeneration and associated loss of function. This suggests that pGSN does not interfere with ET-1 action on its receptors, and corresponds to studies in which GSN knockout mice had larger infarct volume at 22 h even though reductions in CBF during MCAO were not altered [23].

Although this study shows that pGSN in or near an infarct can reduce neuropathology and functional loss due to ischemic stroke, the mechanism by which pGSN mediates protection are not yet clear. One likely mechanism involves actin depolymerization. Upon tissue injury due to glucose/oxygen deprivation (CBF significantly reduced), large amounts of actin can be released from damaged cells into the extracellular space. Since the ionic conditions in the extracellular fluid favor actin polymerization, high amounts of F-actin could be released to potentially increase the viscosity of blood and perturb blood flow through the microvasculature. The actin severing protein gelsolin has a secreted plasma isoform (called plasma gelsolin), which is constitutively active in the high extracellular calcium concentrations of plasma. Plasma gelsolin severs extracellular F-actin to short filaments, and by capping barbed ends, prevents polymerization and favors monomer release. Therefore, pGSN acts as "debris cleaner" limiting inflammation and possibly decrease blood clogging [22]. Another possible mechanism is through anti-apoptotic activity of pGNS. In Jurkat cells, overexpression of gelsolin inhibites cytokine induced apoptosis [30]. It has been reported that gelsolin can form complex with phosphatidylinositol 4, 5-bisphosphate and inhibit capase-3 and -9 activities [31]. In addition, pGSN may also play an important role in regulating inflammation. Future studies will focus on the mechanisms of pGSN protection.

Histone deacetylase inhibitor-mediated neuroprotection against MCAO has been associated with GSN upregulation and reductions in filamentous actin, neither of which was shown to occur in GSN-knockout mice in which the treatment was ineffective [32]. Also, GSN can modulate the actin cytoskeleton regulation of numerous ion channels responsible for elevated cytotoxic intracellular calcium and glutamate excitotoxicity [33-35].

Gelsolin is regulated by phosphatidylinositol 4, 5-bisphosphate (PIP_2_), and contains a lipid signaling binding domain. This domain has been shown to bind to a number of bioactive lipids including lysophosphatidic acid (LPA), lipoteichoic acid (LTA), and lipopolysaccharide (LPS) [36-39]. LPA levels have been shown to be increased in patients suffering ischemic stroke [40]. LPA signaling has also been shown to regulate a number of pro-inflammatory genes [41]. Increasing gelsolin levels during stroke may serve to modulate the inflammatory response thereby offering protection against the inflammation related neurodegeneration following stroke.

Further emphasis of the potential importance of GSN in stroke comes from recent reports that circulating pGSN levels are reduced in ischemic stroke suffers and is highly predictive for first-year mortality from ischemic stroke [37]. Matrix metalloproteinases (MMPs), zinc-containing endopeptidases that participate in both normal and pathological processes, are upregulated during inflammatory conditions [42], including stroke [43]. pGSN is cleaved *in vitro *by MMP-3, MMP-2, MMP-1, MMP-14 and MMP-9 [44] which may be the cause of the severe depletion of pGSN observed in patients who suffer ischemic stroke. Replacing lost pGSN may interrupt pro-inflammatory cascade and result in decreased brain damage.

## Conclusion

The current study offers a proof of principle that delivery of pGSN following ischemic stroke results in neuroprotection and can reduce both sensory and motor deficits that arise following stroke. Future research aimed at characterizing improved delivery, dose response, temporal, safety, pharmacokinetic issues, and physiological mechanisms for further preclinical development of this promising strategy are called for.

## List of abbreviations

ANOVA: Analysis of variance between groups; aCSF: Artificial cerebrospinal fluid; CBF: Cerebral blood flow; CDC: Center for Disease Control and Prevention; ET-1: Endothelin-1; FDA: The U.S Food and Drug Administration; GSN: Gelsolin; pGSN: Plasma gelsolin; LDF: Laser-Doppler flowmetry; LPA: Lysophosphatidic acid; LTP: Lipoteichoic acid; LPS: Lipopolysaccharide; MCA: Middle cerebral artery; MCAO: Middle cerebral artery occlusion; tMCAO: Transient middle cerebral artery occlusion; MMPs: Matrix metalloproteinases; PBF: Phosphate buffered formaldehyde; PBS: Phosphate buffered saline; PU: Perfusion unit; rtPA: Recombinant tissue plasminogen activator; TTC: 2, 3, 5-triphenyltetrazolium chloride.

## Competing interests

The authors declare that they have no competing interests.

## Authors' contributions

HTL and ACH contributed substantially to experimental design, experimental implementation, data analysis, and manuscript preparation. JST contributed to the cerebral perfusion measurement in MG's Lab, JL and JP contributed to the behavioral tests. MAK, JAH and SS conceived of the study, contributed to experimental design, data analysis and revised the manuscript. All authors have read and approved the final version of the manuscript.
